# Editorial Department

**Published:** 1860-10

**Authors:** P. H. Austen

**Affiliations:** Dean.


BALTIMORE, *October* 24, 1860.

*To Dr. A. A. Blandy :*

" Consequent upon the death of our highly esteemed and much lamented colleague, the late Professor Chapin A. Harris, the title of the Chair thus made vacant was " Principles of Dental Science and Practice of Dental Surgery."

The "Principles of Dental Science" have been transferred to the "Chair of Professor," Philip H. Austen and Ferdinand J. S. Gorgas, A. M.. D. D. S., has been elected Professor of the " Practice of Dental Surgery."

P. H. AUSTEN, *Dean.*

